# Identification of Novel Functional Inhibitors of Acid Sphingomyelinase

**DOI:** 10.1371/journal.pone.0023852

**Published:** 2011-08-31

**Authors:** Johannes Kornhuber, Markus Muehlbacher, Stefan Trapp, Stefanie Pechmann, Astrid Friedl, Martin Reichel, Christiane Mühle, Lothar Terfloth, Teja W. Groemer, Gudrun M. Spitzer, Klaus R. Liedl, Erich Gulbins, Philipp Tripal

**Affiliations:** 1 Department of Psychiatry and Psychotherapy, University of Erlangen, Erlangen, Germany; 2 Theoretical Chemistry, Center for Molecular Biosciences, University of Innsbruck, Innsbruck, Austria; 3 Department of Environmental Engineering, Technical University of Denmark, Lyngby, Denmark; 4 Molecular Networks GmbH, Erlangen, Germany; 5 Department of Molecular Biology, University of Duisburg-Essen, Essen, Germany; University of Geneva, Switzerland

## Abstract

We describe a hitherto unknown feature for 27 small drug-like molecules, namely functional inhibition of acid sphingomyelinase (ASM). These entities named FIASMAs (Functional Inhibitors of Acid SphingoMyelinAse), therefore, can be potentially used to treat diseases associated with enhanced activity of ASM, such as Alzheimer's disease, major depression, radiation- and chemotherapy-induced apoptosis and endotoxic shock syndrome. Residual activity of ASM measured in the presence of 10 µM drug concentration shows a bimodal distribution; thus the tested drugs can be classified into two groups with lower and higher inhibitory activity. All FIASMAs share distinct physicochemical properties in showing lipophilic and weakly basic properties. Hierarchical clustering of Tanimoto coefficients revealed that FIASMAs occur among drugs of various chemical scaffolds. Moreover, FIASMAs more frequently violate Lipinski's Rule-of-Five than compounds without effect on ASM. Inhibition of ASM appears to be associated with good permeability across the blood-brain barrier. In the present investigation, we developed a novel structure-property-activity relationship by using a random forest-based binary classification learner. Virtual screening revealed that only six out of 768 (0.78%) compounds of natural products functionally inhibit ASM, whereas this inhibitory activity occurs in 135 out of 2028 (6.66%) drugs licensed for medical use in humans.

## Introduction

Acid sphingomyelinase (ASM, EC 3.1.4.12) is a lysosomal glycoprotein that catalyses the hydrolysis of sphingomyelin into ceramide and phosphorylcholine. Fusion of secretory lysosomes with the cell surface and translocation of lysosomal ASM onto the outer leaflet of the cell membrane plays an important role during stress response [1]. CD95 ligands and cytokines such as tumor necrosis factor-α, interleukin-1 and interferon-γ but also other stimuli including oxidative stress, reactive oxygen and nitrogen species, ionizing radiation, UV-C radiation, heat shock and other agents of stress, injury or infections by HIV or bacteria have been shown to stimulate ceramide production [2]–[7], assumed to be in part due to increased ASM activity. Ceramide, in turn, leads to membrane reorganization and downstream signalling that results in cell activation, very often cell stress or apoptosis. In addition to ASM, at least three other sphingomyelinases have been described in mammalian cells that vary in their pH optimum and cofactor dependency. Although these enzymes and an existing *de novo* synthesis pathway are alternative mechanisms for ceramide generation, activation of ASM itself has been proven to be critical for some cellular responses, such as apoptosis induced by reactive oxygen and nitrogen species [3], chemotherapy drugs such as cisplatin [8], bacteria [5], radiation [9] and CD95 [10]. Furthermore, in contrast to other sphingomyelinases, ASM activity is tightly regulated [11].

Ceramide is further metabolized to sphingosine and sphingosine-1-phosphate by acid ceramidase (AC, EC 3.5.1.23) and sphingosine kinases. While the biological function of sphingosine is largely unknown, sphingosine-1-phosphate has been shown to be involved in cellular differentiation, proliferation and cell migration [12]–[16]. This dynamic balance between ceramide and sphingosine-1-phosphate is referred to as the “ceramide/sphingosine-1-phosphate rheostat” [17]–[19], maintaining the balance between growth and cell death.

ASM is best known for its involvement in Niemann-Pick disease, a lysosomal storage disease due to an inherited enzyme deficiency [20]. Pathological reduction of ASM activity may be caused by mutations in the ASM gene itself. The severity of Niemann-Pick disease correlates with the decrease of ASM activity [21]. However, studies using cells derived from Niemann-Pick disease patients or from ASM knock-out mice revealed that the deficiency of this enzyme might also have beneficial consequences, including anti-apoptotic and cytoprotective effcts. In fact, there is increasing evidence that ASM activation and ceramide accumulation play a central role in the development of common human diseases (reviewed in Smith & Schuchman [22]). Reports have been published of aberrant activation of ASM and/or altered levels of ceramide, for instance, for several psychiatric and neurological disorders such as major depression [23]–[25], morphine antinociceptive tolerance [26], Alzheimer's disease [27]–[29], spinal cord injury [30] and seizure disorder [31].

Therefore, ASM inhibitors hold promise for a number of new clinical therapies and might be used to prevent apoptosis and other negative effects occurring in different disease states such as in ischemia, stroke, Alzheimer's dementia, Parkinson's disease, Huntington's chorea, and of certain infections, in endotoxemia, and in atherosclerosis, and for the therapy of major depressive disorder [23], [32]–[39]. Currently, only few examples of inhibitors directly interacting with ASM are known. These substances include physiological inhibitors of ASM such as phosphatidyl-*myo*-inositol-3,4,5-trisphosphate [40], L-α-phosphatidyl-D-*myo*-inositol-3,5-bisphosphate [41] and non-natural direct inhibitors of ASM, such as SMA-7 [42] and AD2765 [43]. A high throughput screening for direct ASM inhibitors was unsuccessful in finding lead structures [44]. The rational development of compounds that block ASM by direct interaction with the enzyme is difficult, since the crystal structure of the enzyme is not yet available. On the other hand, it has been known since the 1970s that some weak organic bases have the potential to reduce the activity of ASM [23], [45]–[47]. It has been suggested that ASM is bound to intra-lysosomal membranes, and thereby protected against its own proteolytic inactivation. Weak bases, such as desipramine **168** (for numbering of compounds see Table S1), strongly accumulate in acidic intracellular compartments like lysosomes [48], [49], a phenomenon called ‘acid trapping’ and in the case of lysosomes ‘lysosomotropism’. This accumulation of desipramine **168** results in detachment of the ASM from the inner lysosomal membrane [50] and its subsequent inactivation, probably by proteolytic degradation [51]. Weak bases, therefore, do not directly inhibit ASM, but result in a *functional* inhibition of ASM. We have thus proposed the acronym FIASMA for Functional Inhibitor of Acid SphingoMyelinAse [39]. According to this model, functional inhibition of ASM requires high lysosomal concentrations of a weak basic drug.

Previously, we have shown that functional inhibition of ASM is related to high pKa- and high logP-values and have characterized several new FIASMAs, including the antidepressant drugs doxepine **63**, fluoxetine **104**, maprotilin **109**, nortriptyline **114**, paroxetine **118** and sertraline **124** [52]. The aims of the present study were (1) to identify more FIASMAs, (2) to further improve the *in silico* prediction of functional ASM inhibition by developing compact and easily-interpretable models with high internal consistency, (3) to investigate the relationship between permeation of the blood-brain barrier and functional inhibition of ASM and (4) to study the distribution of FIASMAs across different classes of drugs licensed for medical use in humans.

Using the present knowledge about lysosomal accumulation and drug-membrane interaction as prerequisite to functional inhibition of ASM, we developed the following hypotheses: FIASMAs (1) have shared structural and physicochemical properties allowing high lysosomal drug concentrations, compatible with partitioning into the inner surface of the lysosomal membrane and alteration of electrostatic membrane properties. We hypothesize that FIASMAs (2) will be found in different *therapeutic* drug classes and (3) belong to diverse *structural* drug classes. To reach the lysosome, functional inhibition of ASM requires penetration of drugs through biological membranes, comparable to substances crossing the blood-brain barrier (BBB). We therefore hypothesize (4) that FIASMAs also penetrate the BBB and (5) are therefore overrepresented in drugs active in the central nervous system (CNS).

## Results and Discussion

### Identification of novel FIASMAs

We have previously reported functional inhibition of ASM in cell culture for a wide range of compounds at a concentration of 10 µM for 30 min incubation time [52], based on initial experiments with fluoxetine **104**. Using a cell-based simulation model, we have meanwhile realized that very high logP- or pKa-values or the presence of two basic nitrogen atoms leads to slow accumulation kinetics [49]. Therefore, we have also tested longer incubation times for such compounds (Table S1) and found lower residual activities of ASM than previously reported for alverine **40**, astemizole **43**, bepridile **46**, camylofine **50**, cloperastine **56**, dicyclomine **98**, drofenine **64**, mebeverine **110**, mibefradile **74**, pimozide **81** and thioridazine **32** (see Table S1). Several of these substances that were measured [52] as not functionally inhibiting ASM (residual activity of ASM above 50% of control value) are actually FIASMAs when prolonged incubation times are used (alverine **40**, dicyclomine **98**, mebeverine **110**, mibefradile **74**, pimozide **81**). Our results demonstrate that it is important to consider slow lysosomal accumulation kinetics in compounds with very high pKa- and/or very high logP-values and in compounds with two basic nitrogen atoms. In addition to identifying five more of the previously-tested 101 compounds [52] as FIASMAs taking their slower lysosomal accumulation into account (see Table S1: alverine **40**, dicyclomine **98**, mebeverine **110**, mibefradile **74**, pimozide **81**), we have identified 22 other previously unknown FIASMAs out of our new set of 175 small drug-like compounds (residual activity of ASM of ≤50%: aprindine **141**, biperidene **149**, carvedilol **160**, cepharantine **161**, clemastine **170**, clofazimine **172**, conessine **175**, desloratadine **182**, dimebon **189**, emetine **196**, flupenthixol **208**, fluphenazine **209**, fluvoxamine **210**, hydroxyzin **219**, loperamide **227**, mebhydroline **229**, perphenazine **249**, profenamine **258**, sertindole **277**, solasodine **278**, tomatidine **291**, zolantidine **276**). Four of them (dimebon **189**: residual ASM activity 44.1%; fluvoxamine **192**: residual ASM activity 37.4%; hydroxyzin **219**: residual ASM activity 43.0%; mebhydroline **207**: residual ASM activity 41.9%) were classified as FIASMAs albeit with less reliability due to experimental error. To the best of our knowledge, functional inhibition of ASM has not previously been described for any of the compounds presented here. Looking at the whole group of FIASMAs which we experimentally investigated (n = 72, Kornhuber et al. 2008 [52] and Table 1), we noticed some general characteristics. These compounds had moderate to high logP-values (ACD10; mean ± SD: 5.45±1.13; range: 2.03–8.89) and possessed at least one basic nitrogen atom responsible for moderate to high pKa-values (ACD10; mean ± SD: 9.04±1.18; range: 4.81–11.20). This qualifies all FIASMAs as cationic amphiphilic drugs. Most FIASMAs had a molecular weight below 500 (ACD10; mean ± SD: 366.3±85.1; range: 263.4–645.3).

**Table 1:** Newly-identified FIASMAs.

Generic name or substance code	CID	ATC code	FDA status [70]
Alverine 40	3678	A03AX08	Not listed
Mibefradile 74	60663	C08CX01	Not listed
Pimozide 81	16362	N05AG02	Prescription drug
Dicyclomine 98	3042	A03AA07	Prescription drug
Mebeverine 110	4031	A03AA04	Not listed
Aprindine 141	2218	C01BB04	Not listed
Biperidene 149	2381	N04AA02	Prescription drug
Carvedilol 160	2585	C07AG02	Prescription drug
Cepharantine 161	360849	Not listed	Not listed
Clemastine 170	26987	D04AA14	Prescription drug
Clofazimine 172	2794	J04BA01	Prescription drug
Conessine 175	441082	Not listed	Not listed
Desloratadine 182	124087	R06AX27	Prescription drug
Dimebon 189	197033	Not listed	Not listed
Emetine 196	10219	P01AX02	Not listed
Flupenthixol 208	5281881	N05AF01	Not listed
Fluphenazine 209	3372	N05AB02	Prescription drug
Fluvoxamine 210	5324346	N06AB08	Prescription drug
Hydroxyzin 219	3658	N05BB01	Prescription drug
Loperamide 227	3955	A07DA03	Prescription drug
Mebhydroline 229	22530	R06AX15	Not listed
Perphenazine 249	4748	N05AB03	Prescription drug
Profenamine 258	3290	N04AA05	Discontinued drug
Sertindole 277	60149	N05AE03	Not listed
Solasodine 278	442985	Not listed	Not listed
Tomatidine 291	65576	Not listed	Not listed
Zolantidine 306	91769	Not listed	Not listed

See Table S1 for further details and numbering of compounds.

CID = PubChem Compound ID.

### Functional inhibition of ASM is bimodally distributed

**Figure 1:**
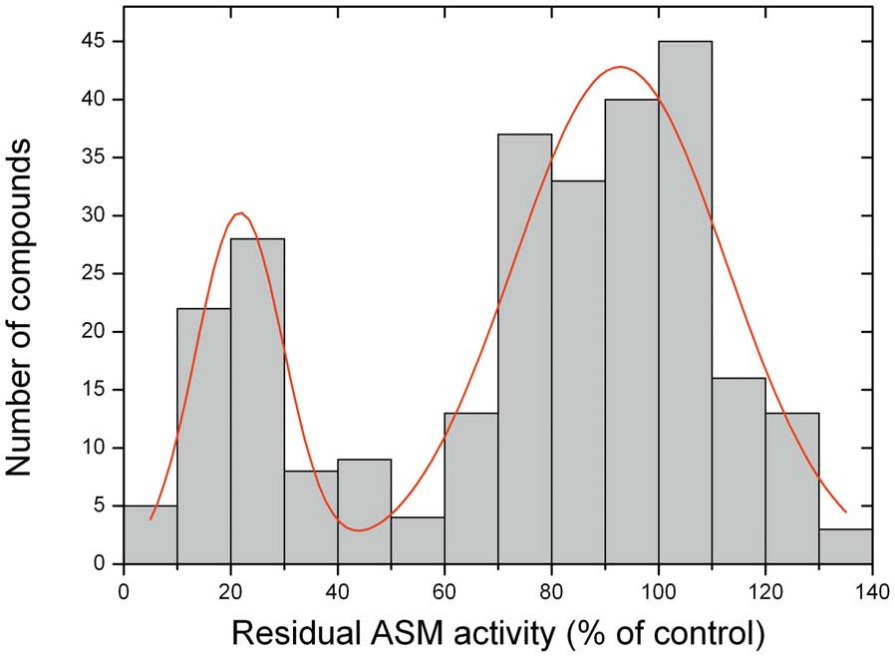
Analyzed compounds show a bimodal distribution with respect to functional inhibition of ASM. The histogram includes results of 276 experimentally investigated compounds (see Table S1). The line represents a Gaussian fit to the two peaks.

Analysis of the whole set of compounds (n = 276), reveals a bimodal distribution of drugs with respect to functional inhibition of ASM (Figure 1) and a significant deviation from the normal distribution (Kolmogoroff-Smirnov: p<0.001), with the lowest occurrence of FIASMAs at a residual activity between 50 and 60%. Both subpeaks of the bimodal distribution did not significantly deviate from a normal distribution. The reason for the bimodal distribution may be the inevitable presence of physicochemical properties that are non-linearly related to lysosomal drug accumulation and that work in a synergistic way. Since our experimentally-tested compounds were not randomly chosen, a selection bias might also contribute to the bimodal distribution.

### Random forest learners provide a good binary prediction of functional inhibition of ASM

We divided the dataset into two groups – active compounds (≤50% residual ASM activity, class 1, n = 72) and inactive compounds (>50% residual ASM activity, class 0, n = 204). Usually, dichotomizing continuous variables results in a number of statistical problems, including a loss of statistical power to detect a relation between a variable and outcome [53]–[55]. In our data, dichotomization is justified by the pronounced bimodal distribution. We performed a *qualitative prediction* of functional inhibition of ASM using the whole set and random forest learners. The prediction accuracy in the set increased with the number of attributes used. A high precision of prediction was reached with n = 4 attributes, which is only marginally improved by adding a fifth descriptor (Table 2). The successful 4-descriptor combination included logPACD10_logWeight, pKa1_plus_pKa2ACD10mod, pKaMA_ACD10 and si_Weight_vsa_pol. These four descriptors were only moderately correlated to each other (Spearman rank correlation r^2^ between −0.598 and 0.314, n = 276) and thus provide complementary information. The descriptors logP and pKa have been discussed most frequently in the context of lysosomal accumulation [48], [49]. Intermediate to high logP values have previously been identified as an important precondition for lysosomal accumulation. However, logPACD10_logWeight ( = logPACD10−½ logMW) is more closely related to the diffusion of small molecules through lipid phases than logP itself and has been used to predict BBB permeation [56], [57]. The descriptor pKa1_plus_pKa2ACD10mod represents the sum of the pKa-values of the most basic and the second-most basic nitrogen atoms and thus takes into account the impact of a second basic functional group. The descriptor pKaMA_ACD10 represents the pKa-value of the most acidic group and thus guarantees that acids are correctly classified as being inactive. vsa_pol is an approximation to the sum of van-der-Waals surface areas of polar atoms and has been described to predict BBB permeability [58]. For this descriptor, both hydrogen bond donors and acceptors count as polar atoms. The size-intensive form of the descriptor was chosen here. The four molecular descriptors thus represent electrostatic (pKa1_plus_pKa2ACD10mod, pKaMA_ACD10), hydrophobic (logPACD10_logWeight), polar (vsa_pol) and steric (weight) properties of the molecules. No further steric descriptors were chosen into the model. The reason for this is probably the low variance of steric descriptors as all of the compounds investigated here are small drug-like molecules. The 4-descriptor model presented here is simple, easy to interpret, has high internal consistency and is meaningful in the context of lysosomal accumulation (Table 2). None of the 276 compounds was incorrectly predicted by the 4-descriptor model.

**Table 2:** Descriptor combinations were selected by a 200-fold bootstrap-validated Youden-index based on a binary random forest learner using the whole experimental set.

		Mean 200fold bootstrap-validation		Final model without validation	
n	Descriptors	Youden-indexMean ± SD	AccuracyMean ± SD	Youden-index	Accuracy
1	logPACD10_logWeight	0.294±0.107	0.736±0.038	0.850	0.949
2	logPACD10_logWeight, pKa1_plus_pKa2ACD10mod	0.567±0.090	0.829±0.031	0.967	0.989
3	logPACD10_logWeight, pKa1_plus_pKa2ACD10mod, pKaMA_ACD10	0.626±0.087	0.852±0.031	0.990	0.993
4	logPACD10_logWeight, pKa1_plus_pKa2ACD10mod, pKaMA_ACD10, si_Weight_vsa_pol	**0.680±0.087**	**0.884±0.030**	**1.000**	**1.000**
5	logPACD10_logWeight, pKa1_plus_pKa2ACD10mod, pKaMA_ACD10, si_Weight_vsa_pol, si_Weight_prot_n_PI	0.714±0.086	0.897±0.027	1.000	1.000

A 4-descriptor combination provided high internal consistency, which is only marginally improved by addition of a fifth descriptor. Bootstrap-validated performance measures are given as mean ± SD values.

Based on a smaller set of experimentally investigated compounds (n = 101) and a decision tree learner we had previously suggested a 3-descriptor model (logPACD10, pKa1ACD10, k) for qualitative prediction of functional inhibition of ASM [52]. Applying this 3-descriptor model on the large experimental dataset available in the present study (n = 276) still results in a reasonable good prediction of functional inhibition of ASM (mean bootstrap-validated Youden-index: 0.575±0.085, mean bootstrap-validated accuracy 0.831±0.033, Youden-index without bootstrap-validation 1.0, accuracy without bootstrap-validation 1.0). However, the descriptor-combinations presented here (Table 2) outperform the previous prediction system. The previously suggested model [52] was now improved in several ways by using advanced molecular descriptors (e.g. logPACD10−½ logMW instead of logPACD10; pKa1ACD10+pKa2ACD10 instead of pKa1ACD10), by using four instead of three molecular descriptors, by using a random forest learner instead of a simple decision tree learner and by using rigorous model validation. This led to a much more precise prediction system.

Whenever a structure-property-activity relationship (SPAR) model is built, there is a probability that the best model is chance correlation. We therefore performed a response permutation test (also known as Y-scrambling [59]–[61]). If a strong correlation remains between the selected descriptors and the randomly permutated response, the significance of the proposed model is suspect. The 4-descriptor model (logPACD10_logWeight, pKa1_plus_pKa2ACD10mod, pKaMA_ACD10, si_Weight_vsa_pol) was recalculated for a randomly reordered response. This procedure was performed 1000 times using different random seeds for permutation, resulting in a mean bootstrapping validated Youden-index of 0.001±0.034 and a mean bootstrapping validated accuracy of 0.675±0.014 for the whole experimental set (n = 276). The performance of the 4-descriptor model applied to the original dataset is therefore more than 15 standard deviations above random (mean accuracy in the whole experimental set = 0.884) indicating a large distance between a random response model and a true response model. The whole set comprised 276 compounds, 204 of them in class 0 (inactive) and 72 in class 1 (active). A model assuming all compounds would belong to class 0 (zero rule model) would have an accuracy of 0.739. The accuracy of the chosen model applied to the original dataset is significantly higher than the zero rule model, whereas the model applied to randomly reordered response values has a performance which is even lower than the zero rule model.

The whole process from data pre-processing to model generation and interpretation was in accordance with the OECD/IOMC guidelines for quantitative structure-activity relationship development [62]. This means that our study had (1) a defined endpoint, (2) an unambiguous algorithm, (3) a defined domain of applicability, (4) appropriate measures of goodness-of-fit, robustness and predictivity and (5) a mechanistic interpretation.

### Additive effect of FIASMAs

**Figure 2:**
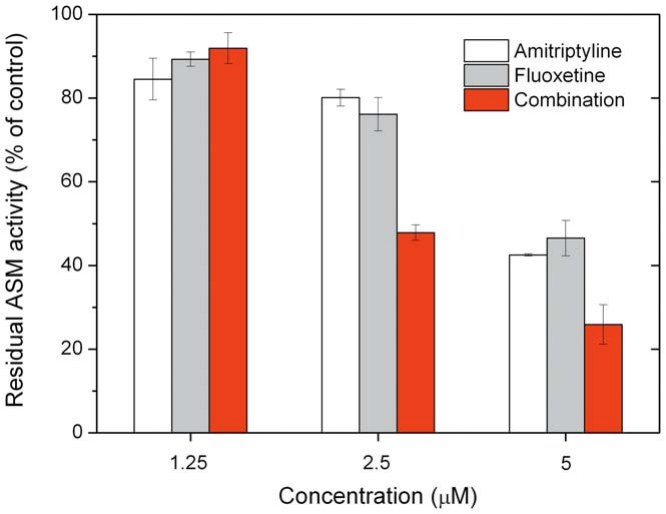
FIASMAs work in an additive way: The combination of a subthreshold-concentration of amitriptyline 6 and fluoxetine 104 (2.5 µM) results in functional inhibition of ASM. This effect is also evident at higher concentrations. Mean values ± SD from 3 experiments are given.

Co-application of structurally diverse FIASMAs results in an additive functional inhibition of ASM, suggesting that these compounds share the same mechanism of inhibition. This is exemplified here by the additive effect of amitriptyline **6** and fluoxetine **104** (Figure 2).

### FIASMAs tend to cross the blood-brain barrier

**Figure 3:**
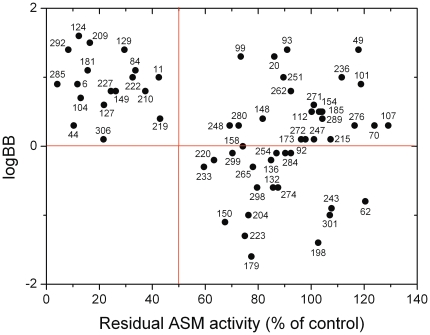
Functional inhibition of ASM appears to be associated with good passive diffusion across the blood-brain barrier. Experimental logBB values were compiled from the literature, with values greater than 0 indicating good blood-brain barrier permeability. Functional inhibition of ASM was experimentally determined in cell culture; residual ASM activity below 50% relative to control cells indicates active compounds. See also results in Table S1.

The blood-brain barrier (BBB) is a selective barrier formed by the endothelial cells that line cerebral microvessels [63]. Tight junctions between adjacent endothelial cells force most molecular traffic across the BBB to take the transcellular route [64]. Small lipophilic agents can diffuse freely through the lipid membranes. Most CNS-active drugs use this transcellular lipophilic pathway. Compounds entering the brain have to cross two membrane barriers (both luminal and abluminal plasma membrane) and the cytoplasm of the endothelial cells in brain capillaries. FIASMAs have to go a similar way through the cell to reach the lysosome: they cross two membrane barriers (plasma membrane of the cell, lysosomal membrane) and the cytoplasm. Therefore an analogy between the BBB and the blood-lysosomal barrier can be assumed. For 64 of the 276 compounds investigated here, we found experimental logBB values (logarithmic ratio between the concentration of a compound in brain and blood) in the literature. When residual ASM activity is plotted against logBB values (Figure 3), it is apparent that functional inhibition of ASM (residual activity ≤50%) is found only for compounds that efficiently cross the BBB (logBB≥0).

### FIASMAs violate Lipinski's Rule-of-Five more often than compounds without effect on ASM

We investigated whether or not our experimentally tested compounds (n = 276, Table S1) are in agreement with Lipinski's Rule-of-Five [65]. While 59 of 204 (28.9%) of compounds lacking a significant effect on ASM activity violated the Lipinski's Rule-of-Five, violation occurred in 37 of 72 (51.4%) of FIASMAs (chi-square test, p = 0.001, Table 3). In most of the FIASMAs violation of the Rule-of-Five was due to high lipophilicity. Nevertheless, FIASMAs are orally active and most of them are probably also CNS-active drugs.

**Table 3:** FIASMAs frequently violate Lipinski's Rule-of-Five.

	Experimentally-determined ASM inhibitory activity	
	FIASMA	Not acting as FIASMA
Rule-of-Five = 0	35	145
Rule-of-Five>0	37	59

Chi-square-test, p = 0.001.

Violation was assumed when there was a contradiction to any of the four rules.

Violation of Lipinski's Rule-of-Five was calculated by ACD10 for all compounds with experimentally-determined ASM activity (n = 276).

### Virtual screening shows that FIASMAs are enriched in groups of drugs acting at excitable cells but are rare among natural products

Most of the clinically approved compounds have been developed without specific goals regarding logP- and pKa-values. Therefore, there should be no remarkable differences in these physicochemical properties between groups of drugs approved for medical use. To get a complete picture of the distribution of functional inhibition of ASM within approved drugs, we used the Anatomical Therapeutic Chemical (ATC) drug classification system recommended by the World Health Organization (WHO). Based on the random forest model described above, we analyzed a total of 2440 compounds distributed across 86 third-level drug groups of the ATC codes (with several substances occurring more than once; 2028 different compounds) by a virtual screening approach and found physicochemical characteristics indicative of functional inhibition of ASM in 157 of the 2440 ATC-listed compounds (6.43%). Since some of the drugs in the ATC system are chemically identical, but have a unique ATC-code because of their different clinical indications, a total of 135 of 2028 unique compounds (6.66%) was predicted to functionally inhibit ASM. The distribution of the 157 functional ASM inhibitors across the 86 pharmacologically relevant therapeutic groups significantly deviated from the expected values (Fisher's exact test, p<0.001), but clustered clearly in a few groups. The multiple test correction on Fisher p-values revealed a significant overrepresentation of FIASMAs in only a few therapeutic groups, namely A03 (drugs for functional gastrointestinal disorders), A15 (appetite stimulants), C08 (calcium channel blockers), D04 (antipruritics), N04 (anti-parkinson drugs), N05 (psycholeptics), N06 (psychoanaleptics) and R06 (antihistamines for systemic use). Since group size of the ATC second level drug groups varies strongly, the p-value has a limited explanatory power. From a biological point of view, groups with a high fraction of FIASMAs may be still of interest, even if the p-value is not significant owing to the small size of the group, such as A04 (antiemetics and antinauseants). One common feature of these ATC subgroups with a significant overrepresentation of FIASMAs is the pharmacodynamic involvement of excitable cells where many FIASMAs appear to act primarily. Furthermore, it is evident, that several of the second-level ATC subgroups with a significant number of FIASMAs belong to the CNS active groups (N04, N05, N06).

In contrast to approved drugs for medical use, we found by virtual screening that FIASMAs are rare in natural products. The molecular descriptors necessary for application of the model were calculable in 787 of the 800 “Pure Natural Products” [66]. Two compounds with quaternary nitrogen atoms were excluded. Then we removed 17 duplicates. In six of the remaining 768 compounds (0.78%) the model indicated functional inhibition of ASM. Five of these predicted compounds were also part of the experimental set (Table S1): cepharanthine **161**, conessine **175**, emetine **196**, solasodine **278** and tomatidine **291** and did indeed show functional inhibition of ASM. The rate of predicted FIASMAs in natural products is much lower than the rate of predicted FIASMAs observed in drugs licensed for medical use in humans (6.66%). The applicability domain of our prediction model is small drug-like molecules. The set of Pure Natural Products differs in chemical space from the small drug-like molecules, meaning that these results have to be interpreted with caution.

### FIASMAs are structurally heterogeneous

Chemical space and structural diversity are important when comparing different sets of compounds. It is not valid to extend results obtained in one set of compounds to other compound collections without taking into account structural diversity. In order to compare the degree of structural diversity between sets of compounds, we have developed a new and simple measure termed “relative structural diversity” (div*_rel_*). This simple measure uses a hierarchical clustering of Tanimoto-coefficients [67] based on 2D-fingerprints (see experimental section) and may be used in addition to more complicated indicators of structural diversity [68]. The div*_rel_* of the FIASMAs (n = 72, div*_rel_* = 0.667) is somewhat lower but still high compared to the div*_rel_* of the whole experimental set (n = 276, div*_rel_* = 0.711). For comparison, div*_rel_* is similar in the set of ATC compounds investigated (n = 2028, div*_rel_* = 0.709) as well as in the set of natural products (n = 768, div*_rel_* = 0.747) (Table 4). Thus, the molecular structure of FIASMAs varies widely, as indicated by the high number of clusters. This is supported by the fact that FIASMAs represent different therapeutic classes and different structural classes as evidenced by their ATC-codes (see Table 1). Our results prove that functional inhibition of ASM does not depend on a specific molecular drug class. Instead of specific structural prerequisites, functional inhibition of ASM requires specific physicochemical characteristics of compounds, resulting in high intra-lysosomal concentrations and partitioning of the drug into the inner leaf of the lysosomal membrane.

**Table 4:** Comparison of the relative structural diversity of the compound sets investigated here.

	n	div*_rel_*
FIASMAs	72	0.667
Whole experimental set	276	0.711
ATC-set	2028	0.709
Pure Natural Products	768	0.747

Structural diversity was estimated by a new measure called relative structural diversity (div*_rel_*, see experimental section). The set of FIASMAs is a subset of the whole experimental set investigated here.

In the current study we used several different compound collections. The experimental set investigated here is showing comparable div*_rel_* values to the ATC set. It is therefore appropriate to apply conclusions from the experimental set onto the larger ATC collection. The set of 72 FIASMAs is a subset of the experimental set, which in turn is largely a subset of the ATC drug library. Therefore, these three sets occupy a comparable region of the chemical space, as all these sets are mainly composed of small drug like molecules. The Pure Natural Products cannot easily be compared to the drug-like molecules as their chemicals space differs.

### Clinical implications of the findings presented here

Most of the newly defined FIASMAs described here are included in the WHO drug list of approved and essential medicines [69] and about half of them appear in the US Food and Drug Administration (FDA)-approved drug list [70] (Table 1). These substances not only possess a low toxicity but also a record of a long-term clinical experience – some have been in use for the last five decades. FIASMAs have a number of favourable properties in the context of clinical application [39], suggesting the potential for rapid advancement into preclinical and/or clinical trials. The therapeutic consequences of functional inhibition of ASM are yet poorly understood, however they may induce a number of clinically desired effects. Ceramide and its metabolite sphingosine-1-phosphate have been shown to antagonistically regulate apoptosis, cellular differentiation, proliferation and cell migration. Inhibition of ASM results in anti-apoptotic, proliferative and anti-inflammatory effects. Furthermore, ASM could play a key role in the pathophysiology of depression and in the action of antidepressant drugs [23]. Inhibitors of ASM therefore hold promise for a number of new clinical therapies. Several examples are given here: Induction of stroke by experimental ischemia of the brain was shown to correlate with an activation of the ASM and a release of ceramide [71], [72]. Importantly, ASM-deficient mice were protected from tissue damage caused by focal cerebral ischemia. Furthermore, the ethanol-induced neuronal cell death is mediated, at least in part, by activation of ASM and generation of ceramide [73]. In addition, the lethal gastrointestinal syndrome, which limits the efficacy of radiation and chemotherapy, apparently results from apoptotic damage of the endothelial cells of the microvasculature of the small intestine. Genetic inactivation of ASM prevents this toxicity [74]. Infection induced by the human immunodeficiency virus type I (HIV-1) serves as a further example. HIV-1 induces a dramatic depletion of CD4+ T cells in infected individuals, finally leading to AIDS (acquired immunodeficiency syndrome). The massive loss of CD4 T-lymphocytes is assumed to result from apoptosis, probably due to enhanced sphingomyelin breakdown and accumulation of intracellular ceramide [75]. Interestingly, HIV-1 infected long-term nonprogressors have less elevated lymphocyte-associated ceramide than subjects with evolving AIDS [76], which is paralleled by a lower frequency of apoptotic CD4 and CD8 cells in long-term nonprogressors compared to patients with AIDS [77]. The endotoxic shock syndrome, which is characterized by systemic inflammation, multiple organ damage, circulatory collapse and death, is caused by disseminated endothelial apoptosis sequentially mediated by TNF and ceramide generation [78]. Blocking of this cascade by ASM inhibitors should be preventive and/or therapeutic against the endotoxic shock syndrome. Finally, an imbalance between ASM and acid ceramidase followed by ceramide accumulation has been demonstrated in the hereditary disease cystic fibrosis [79]. In experimental animals, FIASMAs normalize pulmonary ceramide and inflammation [80]. In a clinical pilot study, amitriptyline **6** led to an improved respiratory function in affected patients [81]. Further examples include the treatment of e.g. Alzheimer's disease, major depression, inflammatory bowel disease [42], liver cell death and anaemia occurring in Wilson disease, and pulmonary edema in acute lung injury [82]. Recently, it has been shown that gilenya (FTY720), a drug licensed for treatment of multiple sclerosis, acts as a FIASMA [83]. This novel mechanism of action may explain at least in part the beneficial effects of gilenya in multiple sclerosis.

AC is a lysosomal enzyme degrading ceramide to sphingosine. Cationic amphiphilic agents like desipramine, chlorpromazine and chloroquine have been shown to result in both, a reduced level of AC protein and in an increased level of cellular ceramide [84], [85]. Despite its potential importance, the influence of medically used drugs on AC has not yet been systematically studied. In a cell culture-based pilot study on more than 100 small drug-like compounds (CM, unpublished), FIASMAs had a significantly stronger AC inhibitory effect than non-FIASMAs. However, the effect on AC was less pronounced compared to their effect on ASM as none of the compounds reduced AC activity to levels below 50%. Moreover, because of the considerably lower enzyme activity of the AC compared to ASM (about 25 fold in cell lysates using fluorescently labelled substrates; CM unpublished), the absolute net effect of potentially inhibitory drugs is expected to be dominated by their influence on ASM. The observation of reduced ceramide levels in the mouse hippocampus after application of amitriptyline or fluoxetine (EG, unpublished results) is in agreement with this hypothesis. Nevertheless, the potential dual effect of small drug-like compounds on ASM as well as AC deserves further research with the aim to identify compounds with specific inhibitory activity for therapeutic use.

### Strengths and limitations of the study

Strength: We identified 27 novel FIASMAs, many of them FDA-approved for clinical use in humans. Model construction was based on a large number of experimentally investigated compounds, and used standard Molecular Operating Environment (MOE) descriptors as well as a multitude of novel hypothesis-driven molecular descriptors, like k, n_pdN, CSA and calculated lysosomal drug concentration (Table 5). We did not use 3D-descriptors, which depend on stereoisomers, preprocessing and energy minimization. Furthermore, we made use of size-intensive descriptors. Remarkably, none of the descriptors of the final model was a standard MOE-descriptor. We applied machine learning algorithms with a low proneness to overfitting (random forest) and performed a rigorous model validation (bootstrap). Our performance measure was well suited for dysbalanced bimodal data (Youden-index). We thus developed a simple model with high internal consistency, high external predictivity (mean bootstrapped accuracy of about 87% in the experimental set), and with a mechanistic interpretation. Finally, we introduce a new measure for comparison of structural heterogeneity between sets of compounds (div*_rel_*).

**Table 5:** List of programmed and calculated descriptors that were used in addition to the standard MOE-descriptors.

Name of the descriptor	Description
AA	Length of the amphiphilic axis [95]
CSA	Cross-sectional area [95]
NOOM	Number of atoms outside the amphiphilic axis [95]
Li	Longest distance from an ionized atom to another atom [95]
Mpc	Longest distance from the atom with the highest partial charge [95]
n_qN	Number of quaternary nitrogen atoms
n_COOH	Number of carboxylic acid functions [133]
n_OpN	Sum of nitrogen and oxygen atoms [134]
n_hal	Number of halogen atoms (based on: Norinder and Haeberlein [134])
n_XpC	Sum of halogen and carbon atoms (based on: Norinder and Haeberlein [134])
n_ion	Number of ionized atoms (based on: Lanevskij et al. [56])
n_PI	Number of positive ionizable groups (based on: Lanevskij et al. [56])
n_pdN	Number of protonized delocalized nitrogen atoms in the N-C = N motif
n_pH	Number of polar hydrogen atoms [135]
n_pol	Number of polar atoms [135]
n_amines	Number of amines
n_pN	number of protonated nitrogen atoms at pH 7
I3	+1 for amines, −1 for acids, otherwise 0 [136]
QMAXneg	Highest negative partial charge [133]
QMAXpos	Highest positive partial charge [133]
QMEANN	Average partial charge in nitrogen atoms
QSUMH	Sum of all partial charges on hydrogen atoms
QSUMO	Sum of all partial charges on oxygen atoms [133]
QMINN	Lowest partial charge on nitrogen atoms
QSUMN	Sum of all partial charges on nitrogen atoms
Qamines	Average partial charge on amines
CLys	Calculated lysosomal concentration [49]
LA	Lipoaffinity [137]
logPACD10_minus_O_N	LogP – number of oxygen and nitrogen atoms [134]
logPACD10_logWeight	logPACD10−½*logWeight [56], [57]
pKa1_plus_pKa2ACD10	pKa1ACD10+pKa2ACD10
pKa1_plus_pKa2ACD10mod	if pKa2ACD10>0: pKa1ACD10 + pKa2ACD10, else: pKa1ACD10
si_Weight-Descriptors	Size-intensive descriptors [96] using molecular weight as divisor

Limitations: The chemoinformatic model presented here is valid for small drug-like molecules only. The model allows the qualitative prediction only. The effect of a substance on ASM activity under therapeutic conditions *in vivo* depends on the interplay between compound-, treatment- (such as dose and application route) and organism-related variables. Future studies should clarify whether or not functional inhibition of ASM occurs at therapeutic concentrations of the drugs investigated here.

### Conclusions

Functional inhibitors of ASM (FIASMAs) are an important class of drugs with newly emerging broad clinical applications. In the present study we have identified 27 novel FIASMAs. Based on a large set of experimental data, we have developed a chemoinformatic model for the accurate prediction of functional inhibition of ASM. Furthermore, we describe important properties of FIASMAs, such as BBB permeability.

## Materials and Methods

### Set of compounds

In addition to the set of 101 compounds previously experimentally investigated with regard to functional inhibition of ASM [52], we selected 175 small organic, drug-like compounds or natural products. The selection aimed at obtaining compounds with high structural diversity, with diversity regarding strength and number of acidic and basic functional groups, and diversity regarding logP values. Basic lipophilic compounds were overrepresented in order to obtain a sufficient number of FIASMAs and in order to avoid a greatly unbalanced class distribution for classification tasks. Of the total set of 276 compounds (Table S1) with a mean molecular weight of 359.9 (88.2–847.0), 23 were acids with a most acidic pKa-value as estimated by ACD10 [86] below 10 and no relevant basic group (“acid”) and 155 were weak bases with a most basic pKa-value≥3 and neither another relevant basic nor any relevant acidic group (“monobase”). Further 22 compounds possessed both a basic nitrogen atom with a pKa-value≥3 and an acidic group with a pKa-value below 10 (“zwitter”). Additional 45 compounds contained two basic nitrogen atoms both with pKa-values≥3 and no relevant acidic group (“bibase”). The remaining 31 compounds either had no ionisable functional group at all (n = 9) or had pKa-values outside the biologically relevant range (n = 22).

### Structure entry

Molecular structures were obtained from the PubChem-Project page [87], [88] (Table S1).

### Chemicals

Aclacinomycin-A **133**, acrivastine **134**, amorolfine **139**, clonidine **173**, lynestrenol **228**, montelukast **239** and rolipram **271** were purchased from Biotrend Chemikalien (Köln, Germany); phenylmethylsulfonyl fluoride **253** and S-methylisothiourea **274** from Calbiochem (Merck, Darmstadt, Germany); allylestrenol **135**, atovaquone **142**, barnidipine **146**, butenafine **155**, cepharanthine **161**, chlorquinaldol **166**, clemastine **170**, dienestrol **187**, dutasteride **195**, nelfinavir **243**, solasodine **278** and vinblastine **299** from Chemos (Regenstauf, Germany) and dimebon **189** from Aurora Feinchemie (Graz, Austria). Apomorphin **140**, doxorubicin **193**, phenserine **251**, pranlukast **257**, raloxifene **266** and zolantidine (SKB41) **306** were obtained from Tocris (Bristol, UK) and aprindine **141**, biperidene **149**, buclicine **152**, buspiron **154**, diosmin **190**, emetine **196**, encainide **197**, fosinopril **211**, hydroxyzin **219**, ropinirole **272**, thiocarlide **287**, tiagabine **288** and zafirlukast **305** from VWR (Darmstadt, Germany). Quetiapine **264** was a gift of AstraZeneca (Wedel, Germany). All other substances investigated experimentally were obtained from Sigma-Aldrich (Munich, Germany). All compounds were used in the highest purity available.

### Cell culture

Human brain neuroglioma H4-cells were purchased from Promochem (Wesel, Germany). H4-cells were cultured as described previously [52]. Only cells tested negative for mycoplasma infection were used in the experiments.

### Kinetics of lysosomal accumulation

Usually, 30 min of incubation of H4-cells with the test drug was sufficient to induce functional inhibition of ASM. Due to slow accumulaion kinetics some compounds required a longer incubation time. However, prolonged incubation with some compounds (astemizole **43**, mibefradil **74**, penfluridol **79**, fluoxetine **104**, sertindole **249**, thioridazine **32**) resulted in cell death. We therefore aimed at a minimal but still sufficient incubation time for all compounds, which was estimated using a previously developed numerical single cell model [49]: Accumulation of molecules in the cell by diffusion from the external solution into the cytosol, lysosome and mitochondria was calculated with the Fick-Nernst-Planck-equation. The cell model considers the diffusion of neutral and ionic molecules across biological membranes, dissociation into mono- or bivalent ions, adsorption to lipids, and electrical attraction or repulsion. We used the parameters of the standard model [49] as well as the logP-, most basic and most acidic pKa-values as calculated by ACD10 [86] (Table S1). Four different models (“monoacid”, “monobase”, “bibase” and “zwitter”) implemented in Excel sheets were used for kinetic calculations (model version: July 2009). There was no amphoteric compound in our dataset. In the absence of pKa-values in the relevant regions, the monoacid model was used with a pKa-value of 15.

### Experimental determination of ASM activity

The activity of ASM was determined in whole cell lysates, as previously described [89]. We aimed to develop a qualitative (binary) SPAR model. Therefore, we did not determine IC50 values, but we determined whether or not a compound acts as functional inhibitor of ASM. Thus, we used a single high concentration of a compound and a sufficient incubation time, which depends on the pharmacokinetic properties of the compound. After the substance was added to the growth medium at a final concentration of 10 µM, cells were kept at 37°C in a humidified atmosphere at 8.5% CO_2_ for 30 minutes (DMEM: pH 7.5). Compounds with predicted slow lysosomal accumulation kinetics [49] were also investigated at longer incubation times (Table S1). Results are given as residual ASM activity (%) normalized to control cells treated with the solvent alone and represent mean values of three independent experiments, each with a standard deviation of approx. 16%. Because of the bimodal distribution of functional inhibition of ASM (see below), a residual ASM activity ≤50.0% in H4-cells was rated as positive.

### Statistical analysis

Deviation from normal distribution (two-sided Kolmogoroff-Smirnov) and chi-square statistics were computed using PASW (Version 18, Chicago, Illinois). One-sided Fisher's exact probability test with Benjamini-Hochberg [90] correction for multiple testing was computed using R software (version version 2.7.0) [91].

### Computation of molecular descriptors

Molecular descriptors were calculated by the ACD/Log*D* Suite, version 10 [86] (the two most basic pKa-values, the most acidic pKa-value, logP-value), by visual inspection of the molecules [number of heavy atoms at the most basic nitrogen atom (k [52])] and by MOE 2009.10 [92]. The lysosomal concentration of each drug was calculated by a previously developed numerical single cell model [49] (see above) and was also used as a molecular descriptor (CLys). It has previously been suggested that the lowest cross-sectional area of a molecule perpendicular to the amphiphilic axis determines the insertion of a molecule into a biomembrane and thereby its passive diffusion across the BBB [93], [94]. The lowest cross-sectional area (CSA, Table 5) of the molecules perpendicular to the amphiphilic axis in the membrane bound conformation and related molecular descriptors were calculated as previously described [95]. A simple division of molecular descriptors by chemical sample size creates size-intensive descriptors which were shown to result in more compact and more stable models [96]. We used molecular weight (MW) to calculate a size-intensive form of every molecular descriptor. Furthermore, we used an MW-based correction of logP-values (logP−½*logWeight [56], [57]). Further information on these descriptors is available in Table 5 and in the literature [59], [97].

### Data pre-processing

Data pre-processing was performed using RapidMiner 5.1 [98]. Features with very low variance were eliminated. Missing values for the most basic pKa-values were substituted by 0, missing most acidic pKa-values were substituted by 15, missing values for k were substituted by 12. Otherwise, we used only molecular descriptors that were available for all compounds and thereby obtained a total of 708 molecular descriptors. The presence of irrelevant and redundant attributes may reduce the performance of learners such as decision tree and random forest [99]. Therefore, before applying random forest learners we reduced the dimensionality of the data by applying feature selection methods. We used both a hypothesis-driven and a hypothesis-free feature selection approach [100], [101] to select descriptors with potential relevance. For the hypothesis-free selection, attributes were chosen by at least one of the following methods: wrapper [102], relief [103], [104], correlation based weighting, information gain weighting, standard deviation based weighting, support vector machine based weighting and rule based weighting. We thus ended up with about 100 molecular descriptors used for random forest learners.

### Model construction

Models were generated using machine learning algorithms [105], [106] provided by RapidMiner 5.1 [98]. The whole experimental set was used, since examples are too precious to waste during model generation [107]. A modified beam-search [108] served to identify high-performing single attributes or attribute combinations. Models were validated by bootstrapping (n = 200). Because of the unbalanced class distribution, the Youden-Index [109]–[111] (sensitivity + specificity−1) was chosen as primary performance measure evaluating models based on two-class learners. We used a random forest learner [112] for binary classification. In contrast to a simple decision tree learner, random forest learners are more resistant against overfitting and result in more stable models, meaning that small changes in the training set do not lead to completely different models [113], [114], and lead to better predictivity on unknown compounds. The parameters used were: ntree = 51 (number of trees to build), nodesize = 1 (size of the terminal node of the trees in a random forest), mtry = log(m)+1 (number of attributes randomly selected at each node, where m = number of attributes available), allowed maximal depth of the trees = 8.

Whenever a structure activity relations (SAR) model is built, there is a probability that the best model is chance correlation. We used the response permutation test, also known as Y-scrambling [59]–[61], [115] to detect random effects.

### Relative structural diversity of the set of compounds used

In order to estimate the structural diversity of our investigated sets of compounds, we performed a hierarchical clustering based on Tanimoto coefficients [67] for fingerprints based on tagged graph triangles (TGT) [116], [117]. We calculated the number of clusters at 0.85 similarity measured by Tanimoto coefficients and divided it by the total number of compounds in the test set. We term this measure “relative structural diversity” (div*_rel_*).
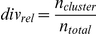



Higher values for div*_rel_* indicate higher structural diversity. The values for div*_rel_* can vary from just above zero (when there is only one cluster) to 1 (when each compound defines a separate cluster). For our analysis, we have used the structural TGT keys [116], [117] consisting of 2D pharmacophore features with atom types and distances.

### Violation of Lipinski's Rule-of-Five

Lipinski's Rule-of-Five [65] identifies compounds with properties that would likely make them an orally active drug in humans. The set of four rules describes molecular properties important for a drug's pharmacokinetic properties in the human organism, including its absorption, distribution, metabolism, excretion and toxicity (ADME/Tox). Violation of Lipinski's Rule-of-Five was calculated by ACD10 logD Suite. Violation was assumed when there was a contradiction to any of the four rules.

### Data on passive diffusion across the blood-brain barrier

The most common value describing permeability across the blood-brain barrier (BBB) is the logBB. It is defined as the logarithmic ratio between the concentration of a compound in brain and blood. LogBB values below zero indicate poor penetration of the BBB. LogBB values were compiled from a number of published papers [58], [118]–[128].

### Distribution of FIASMAs across the therapeutic and structural classes of the ATC system

The five-level ATC drug classification system [69], [129], [130] was developed in the early 1970s and provides a comprehensive and logical classification system for categorizing pharmaceutical ingredients approved for medical use in humans. While the first level indicates the main anatomical target of the compound, the second level codes for the pharmacologically-relevant therapeutic main group. The third level indicates the pharmacological subgroup and the fourth the chemical subgroup. The fifth level represents the chemical substance, namely the actual drug entity. On the first level, the code comprises 14 anatomical regions ranging from A (Alimentary tract and metabolism) to V (Various). On the second level, the code consists of 86 pharmacologically-relevant therapeutic groups. Molecular structures of the drugs in their corresponding free base/acid forms were obtained from the SuperDrug database [131]. Export was performed in May 2007, based on the 2005 version of the ATC system. Since we analyzed molecular descriptors of defined molecules, our approach excluded drugs which are entire plants, extracts, drug mixtures, colloids, biopolymers, resins, large peptides and inorganic substances like metals and simple salts. Furthermore, we excluded compounds with a quaternary nitrogen atom, as they do not cross biological membranes via passive diffusion [56]. All compounds were analyzed by their appearance in the ATC system, i.e. substances with multiple effects and different therapeutic indications or different enantiomeric forms were found more than once within the system. In order to determine the distribution of drugs across the ATC system, we used the second level of the 2005 version. Uneven distribution of FIASMAs was tested by a contingency table and one-sided Fisher test. Within this contingency table, the multiple test correction (Benjamini-Hochberg [90]) identified therapeutic groups with a significantly enriched fraction of FIASMAs [132].

### Distribution of FIASMAs across natural products

Molecular structures of “Pure Natural Products” were downloaded from MicroSourceDiscovery [66] and analysed using the random forest model based on the whole experimental set. This set of “Pure Natural Products” consisted of 800 alkaloids, flavonoids, sterols/triterpenes, diterpenes/sesquiterpenes, benophenones/chalcones/stilbenes, limonoids/quassinoids and chomones/courmarines, benzofurans/benzopyrans, rotenoids/xanthones, carbohydrates and benztropolones/depsides/depsidones. Compounds with a quaternary nitrogen atom and duplicates were again excluded from this analysis.

## Supporting Information

Table S1 Compounds investigated in this study.
(DOC)
