# Supplementary material for: Identification of Novel Functional Inhibitors of Acid Sphingomyelinase
Source: PLoS One. 2011 Aug 31;6(8):e23852. doi: 10.1371/journal.pone.0023852 (PMC3166082; doi:10.1371/journal.pone.0023852)
Supplement: Table S1 — Compounds investigated in this study. (DOC) [file pone.0023852.s001.doc]

**Supporting Information: Table S1**

The whole set of compounds investigated here (n=276) is based on a previous publication (Kornhuber et al. 2008 [1], n=101) combined with an additional set of compounds (n=175). The numbering of compounds is identical to the previous publication for the first 101 compounds. CID = PubChem Compound ID. Set: 1 = experimental values from Kornhuber et al. 2008 [1], 2 = corrected experimental values of the previously published compounds; 3 = newly investigated compounds, Acid/Base: 1 = monobase, 2 = monoacid, 3 = bibase, 4 = zwitter. Mean residual activity of ASM is given as % of the corresponding control values.

1. Kornhuber J, Tripal P, Reichel M, Terfloth L, Bleich S et al. (2008) Identification of new functional inhibitors of acid sphingomyelinase using a structure-property-activity relation model. J Med Chem 51: 219-237

| **Compound #** | **Name** | **Set** | **CID** | **Acid/Base** | **Incubation time (h)** | **residual ASM activity** |
| --- | --- | --- | --- | --- | --- | --- |
| **2008-Paper** |  |  |  |  |  |  |
| 6 | Amitriptyline | 1 | 2160 | 1 | 0.5 | 11.7 |
| 11 | Chlorpromazine | 1 | 2726 | 1 | 0.5 | 42.4 |
| 12 | Clomipramine | 1 | 2801 | 1 | 0.5 | 21.8 |
| 20 | Haloperidol | 1 | 3559 | 1 | 0.5 | 86.1 |
| 25 | Perhexiline | 1 | 4746 | 1 | 0.5 | 8.5 |
| 32 | Thioridazin | 2 | 5452 | 1 | 6 | 10.4 |
| 34 | Trihexyphenidyl | 1 | 5572 | 1 | 0.5 | 83.9 |
| 35 | Trimipramine | 1 | 5584 | 1 | 0.5 | 13.8 |
| 39 | Alaproclate | 1 | 6603696 | 1 | 0.5 | 92.3 |
| 40 | Alverine | 2 | 3678 | 1 | 24 | 21.7 |
| 41 | Amlodipine | 1 | 2162 | 1 | 0.5 | 12.0 |
| 42 | Antazoline | 1 | 2200 | 3 | 0.5 | 100.9 |
| 43 | Astemizole | 2 | 2247 | 3 | 6 | 14.3 |
| 44 | Benfluorex | 1 | 2318 | 1 | 24 | 69.7 |
| 45 | Benztropine | 1 | 6832 | 1 | 0.5 | 12.7 |
| 46 | Bepridil | 2 | 2351 | 3 | 24 | 27.1 |
| 47 | Bromhexine | 1 | 2442 | 1 | 48 | 102.8 |
| 48 | Bromopride | 1 | 2446 | 1 | 0.5 | 90.5 |
| 49 | Bupropion | 1 | 444 | 1 | 0.5 | 117.9 |
| 50 | Camylofin | 2 | 5902 | 3 | 24 | 21.7 |
| 51 | Carbetapentane | 1 | 2562 | 1 | 0.5 | 88.7 |
| 52 | Cinnarizine | 1 | 1547484 | 1 | 48 | 48.9 |
| 53 | Cisapride | 1 | 2769 | 1 | 0.5 | 101.3 |
| 54 | Clebopride | 1 | 2780 | 1 | 0.5 | 102.0 |
| 55 | Clomiphene | 1 | 1548955 | 1 | 0.5 | 13.0 |
| 56 | Cloperastine | 2 | 2805 | 1 | 24 | 26.7 |
| 57 | Cloricromen | 1 | 68876 | 1 | 0.5 | 119.5 |
| 58 | Cyclazocine | 1 | 19143 | 1 | 0.5 | 123.3 |
| 59 | Cyclobenzaprine | 1 | 2895 | 1 | 0.5 | 26.2 |
| 60 | Cyproheptadine | 1 | 2913 | 1 | 0.5 | 22.2 |
| 61 | Dilazep | 1 | 3074 | 3 | 24 | 41.6 |
| 62 | Domperidone | 1 | 3151 | 1 | 0.5 | 120.4 |
| 63 | Doxepin | 1 | 667477 | 1 | 0.5 | 46.6 |
| 64 | Drofenine | 2 | 3166 | 1 | 24 | 20.8 |
| 65 | Etomidate | 1 | 36339 | 1 | 0.5 | 109.1 |
| 66 | Fipexide | 1 | 3351 | 1 | 0.5 | 121.3 |
| 67 | Flunarizine | 1 | 941361 | 1 | 48 | 32.7 |
| 68 | Fluspirilene | 1 | 3396 | 1 | 0.5 | 103.9 |
| 69 | Harmine | 1 | 5280953 | 1 | 0.5 | 92.1 |
| 70 | Lidocaine | 1 | 3676 | 1 | 0.5 | 123.9 |
| 71 | Lofepramine | 1 | 3947 | 1 | 24 | 19.2 |
| 72 | Loratadine | 1 | 3957 | 1 | 48 | 48.5 |
| 73 | Mecamylamine | 1 | 4032 | 1 | 0.5 | 112.1 |
| 74 | Mibefradil | 2 | 60663 | 3 | 6 | 20.8 |
| 75 | Mifepristone | 1 | 55245 | 1 | 24 | 126.7 |
| 76 | Noscapine | 1 | 4544 | 1 | 0.5 | 114.8 |
| 77 | Oxybutynine | 1 | 4634 | 1 | 48 | 63.4 |
| 78 | Papaverine | 1 | 4680 | 1 | 24 | 98.3 |
| 79 | Penfluridol | 1 | 33630 | 1 | 6 | 22.0 |
| 80 | Pimethixene | 1 | 4822 | 1 | 24 | 16.5 |
| 81 | Pimozide | 2 | 16362 | 1 | 24 | 30.6 |
| 82 | Pridinol | 1 | 4904 | 1 | 0.5 | 94.3 |
| 83 | Procyclidine | 1 | 4919 | 1 | 0.5 | 85.4 |
| 84 | Promazine | 1 | 4926 | 1 | 0.5 | 33.6 |
| 85 | Protriptyline | 1 | 4976 | 1 | 0.5 | 12.7 |
| 86 | Ritanserin | 1 | 5074 | 1 | 48 | 51.2 |
| 87 | Sibutramin | 1 | 5210 | 1 | 0.5 | 63.2 |
| 88 | Tetracaine | 1 | 5411 | 1 | 0.5 | 79.1 |
| 89 | Tofisopam | 1 | 5502 | 1 | 0.5 | 96.1 |
| 90 | Vinpocetine | 1 | 443955 | 1 | 24 | 90.5 |
| 91 | Amantadine | 1 | 2130 | 1 | 24 | 92.3 |
| 92 | Atropine | 1 | 174174 | 1 | 24 | 92.4 |
| 93 | Bromperidol | 1 | 2448 | 1 | 0.5 | 90.9 |
| 94 | Chlorprothixene | 1 | 667467 | 1 | 0.5 | 22.4 |
| 95 | Citalopram | 1 | 2771 | 1 | 0.5 | 79.9 |
| 96 | Clozapine | 1 | 2818 | 3 | 48 | 94.3 |
| 97 | Dextromethorphan | 1 | 5360696 | 1 | 0.5 | 82.7 |
| 98 | Dicyclomine | 2 | 3042 | 1 | 24 | 18.6 |
| 99 | Diphenhydramine | 1 | 3100 | 1 | 0.5 | 73.4 |
| 100 | Diphenylpyralin | 1 | 3103 | 1 | 0.5 | 84.1 |
| 101 | Donepezil | 1 | 3152 | 1 | 0.5 | 118.8 |
| 102 | Fendiline | 1 | 3336 | 1 | 0.5 | 25.2 |
| 103 | Flavoxate | 1 | 3354 | 1 | 24 | 104.9 |
| 104 | Fluoxetine | 1 | 3386 | 1 | 0.5 | 13.0 |
| 105 | Flupirtine | 1 | 53276 | 1 | 0.5 | 124.1 |
| 106 | Ketotifen | 1 | 3827 | 1 | 0.5 | 105.1 |
| 107 | Lamotrigine | 1 | 3878 | 1 | 0.5 | 129.1 |
| 108 | Lercanidipine | 1 | 65866 | 1 | 48 | 75.4 |
| 109 | Maprotiline | 1 | 4011 | 1 | 0.5 | 13.5 |
| 110 | Mebeverine | 2 | 4031 | 1 | 24 | 31.8 |
| 111 | Memantine | 1 | 4054 | 1 | 0.5 | 75.6 |
| 112 | Mirtazapine | 1 | 4205 | 1 | 0.5 | 100.2 |
| 113 | Norfluoxetine | 1 | 4541 | 1 | 0.5 | 22.5 |
| 114 | Nortriptyline | 1 | 4543 | 1 | 0.5 | 13.3 |
| 115 | Orphenadrine | 1 | 4601 | 1 | 0.5 | 74.6 |
| 116 | Oxymetazoline | 1 | 4636 | 1 | 0.5 | 125.0 |
| 117 | Oxyphencyclimine | 1 | 4642 | 1 | 0.5 | 121.9 |
| 118 | Paroxetine | 1 | 43815 | 1 | 0.5 | 31.7 |
| 119 | Pipamperone | 1 | 4830 | 3 | 0.5 | 113.2 |
| 120 | Promethazin | 1 | 4927 | 1 | 0.5 | 32.2 |
| 121 | Pyrilamine | 1 | 4992 | 3 | 0.5 | 103.7 |
| 122 | Reboxetine | 1 | 65856 | 1 | 0.5 | 105.3 |
| 123 | Selegeline | 1 | 26757 | 1 | 0.5 | 83.0 |
| 124 | Sertraline | 1 | 68617 | 1 | 0.5 | 12.3 |
| 125 | Suloctidil | 1 | 5354 | 1 | 0.5 | 21.9 |
| 126 | Sulpiride | 1 | 5355 | 4 | 24 | 90.9 |
| 127 | Terfenadine | 1 | 5405 | 1 | 0.5 | 21.8 |
| 128 | Tramadol | 1 | 33741 | 1 | 0.5 | 103.9 |
| 129 | Triflupromazine | 1 | 5568 | 1 | 0.5 | 29.5 |
| 130 | Triprolidine | 1 | 688585 | 3 | 0.5 | 117.9 |
| 131 | Xylometazoline | 1 | 5709 | 1 | 0.5 | 83.8 |
| **New set** |  |  |  |  |  |  |
| 132 | Acetylsalicylic-acid | 3 | 2244 | 2 | 0.5 | 85.6 |
| 133 | Aclacinomycin_A | 3 | 451415 | 4 | 0.5 | 108.4 |
| 134 | Acrivastine | 3 | 5284514 | 4 | 6 | 93.6 |
| 135 | Allylestrenol | 3 | 235905 | 2 | 24 | 82.3 |
| 136 | Alprenolol | 3 | 2119 | 1 | 0.5 | 84.9 |
| 137 | Ambroxol | 3 | 2132 | 1 | 0.5 | 78.4 |
| 138 | Amiodarone | 3 | 2157 | 1 | 24 | 14.5 |
| 139 | Amorolfine | 3 | 2168 | 1 | 24 | 57.1 |
| 140 | Apomorphin | 3 | 2215 | 4 | 0.5 | 93.5 |
| 141 | Aprindine | 3 | 2218 | 3 | 6 | 27.5 |
| 142 | Atovaquone | 3 | 74989 | 2 | 0.5 | 104.4 |
| 143 | AY9944 | 3 | 9705 | 3 | 0.5 | 22.1 |
| 144 | Azaperone | 3 | 15443 | 3 | 0.5 | 71.4 |
| 145 | Azithromycin | 3 | 447043 | 3 | 24 | 74.8 |
| 146 | Barnidipine | 3 | 65884 | 1 | 6 | 70.6 |
| 147 | Benzbromarone | 3 | 2333 | 2 | 6 | 99.7 |
| 148 | Betaxolol | 3 | 2369 | 1 | 0.5 | 81.7 |
| 149 | Biperidene | 3 | 2381 | 1 | 24 | 26.2 |
| 150 | Bromocriptine | 3 | 31101 | 2 | 24 | 67.5 |
| 151 | Brompheniramine | 3 | 6834 | 3 | 0.5 | 72.7 |
| 152 | Buclicine | 3 | 6729 | 1 | 6 | 62.7 |
| 153 | Bupivacain | 3 | 2474 | 1 | 0.5 | 82.3 |
| 154 | Buspiron | 3 | 2477 | 3 | 6 | 103.0 |
| 155 | Butenafine | 3 | 2484 | 1 | 24 | 79.4 |
| 156 | Butorphanol | 3 | 5361092 | 1 | 0.5 | 69.8 |
| 157 | Calcipotriol | 3 | 5288783 | 2 | 6 | 85.2 |
| 158 | Carbamazepine | 3 | 2554 | 1 | 0.5 | 74.2 |
| 159 | Carbenoxolone | 3 | 636403 | 2 | 6 | 105.6 |
| 160 | Carvedilol | 3 | 2585 | 1 | 24 | 22.4 |
| 161 | Cepharanthine | 3 | 360849 | 3 | 6 | 9.2 |
| 162 | Chloropyramine | 3 | 25295 | 3 | 0.5 | 71.4 |
| 163 | Chloroquine | 3 | 2719 | 3 | 0.5 | 63.3 |
| 164 | Chlorotrianisene | 3 | 11289 | 2 | 24 | 88.6 |
| 165 | Chlorpheniramine | 3 | 2725 | 3 | 0.5 | 77.6 |
| 166 | Chlorquinaldol | 3 | 6301 | 4 | 0.5 | 91.2 |
| 167 | Cibenzoline | 3 | 2747 | 1 | 0.5 | 113.1 |
| 168 | Cilnidipine | 3 | 5282138 | 1 | 6 | 101.1 |
| 169 | Clarithromycin | 3 | 84029 | 1 | 0.5 | 90.3 |
| 170 | Clemastine | 3 | 26987 | 1 | 24 | 12.6 |
| 171 | Clenbuterol | 3 | 2783 | 1 | 0.5 | 105.1 |
| 172 | Clofazimine | 3 | 2794 | 1 | 48 | 23.7 |
| 173 | Clonidine | 3 | 2803 | 1 | 6 | 96.2 |
| 174 | Colchicine | 3 | 6167 | 2 | 0.5 | 97.7 |
| 175 | Conessine | 3 | 441082 | 3 | 24 | 20.8 |
| 176 | Cyclofenil | 3 | 2898 | 2 | 6 | 101.7 |
| 177 | Cyclopentolate | 3 | 2905 | 1 | 0.5 | 93.9 |
| 178 | Cypermethrin | 3 | 2912 | 2 | 6 | 100.5 |
| 179 | D_Mannitol | 3 | 6251 | 2 | 0.5 | 77.4 |
| 180 | Daunorubicin | 3 | 30323 | 4 | 0.5 | 78.4 |
| 181 | Desipramine | 3 | 2995 | 1 | 0.5 | 15.6 |
| 182 | Desloratadine | 3 | 124087 | 3 | 0.5 | 21.9 |
| 183 | Desogestrel | 3 | 40973 | 2 | 6 | 105.3 |
| 184 | Dexamethasone | 3 | 5743 | 2 | 0.5 | 86.3 |
| 185 | Diazepam | 3 | 3016 | 2 | 0.5 | 104.2 |
| 186 | Dibenzosuberane | 3 | 70029 | 2 | 6 | 96.7 |
| 187 | Dienestrol | 3 | 667476 | 2 | 24 | 88.3 |
| 188 | Diltiazem | 3 | 39186 | 1 | 0.5 | 136.5 |
| 189 | Dimebon | 3 | 197033 | 3 | 48 | 44.1 |
| 190 | Diosmin | 3 | 5281613 | 2 | 6 | 98.5 |
| 191 | Dirithromycin | 3 | 6473883 | 3 | 0.5 | 86.0 |
| 192 | Disopyramide | 3 | 3114 | 3 | 0.5 | 129.6 |
| 193 | Doxorubicin | 3 | 1691 | 4 | 0.5 | 100.3 |
| 194 | Droperidol | 3 | 3168 | 1 | 0.5 | 78.7 |
| 195 | Dutasteride | 3 | 152945 | 2 | 24 | 61.0 |
| 196 | Emetine | 3 | 10219 | 3 | 24 | 0.4 |
| 197 | Encainide | 3 | 48041 | 1 | 6 | 107.1 |
| 198 | Enoxolone | 3 | 10114 | 2 | 6 | 102.7 |
| 199 | Epinastine | 3 | 3241 | 1 | 0.5 | 102.4 |
| 200 | Erythromycine | 3 | 8233 | 1 | 0.5 | 131.7 |
| 201 | Fenfluramine | 3 | 3337 | 1 | 0.5 | 79.2 |
| 202 | Fenofibrate | 3 | 3339 | 2 | 24 | 102.2 |
| 203 | Fenspiride | 3 | 3344 | 1 | 0.5 | 115.5 |
| 204 | Fexofenadine | 3 | 3348 | 4 | 6 | 76.3 |
| 205 | FG7142 | 3 | 4375 | 1 | 0.5 | 103.1 |
| 206 | Flecainide | 3 | 3356 | 1 | 0.5 | 138.3 |
| 207 | Flufenamic acid | 3 | 3371 | 2 | 0.5 | 119.2 |
| 208 | Flupenthixol | 3 | 5281881 | 3 | 0.5 | 18.2 |
| 209 | Fluphenazine | 3 | 3372 | 3 | 0.5 | 16.5 |
| 210 | Fluvoxamine | 3 | 5324346 | 1 | 0.5 | 37.4 |
| 211 | Fosinopril | 3 | 55891 | 2 | 0.5 | 97.6 |
| 212 | Fulvestrant | 3 | 104741 | 2 | 6 | 84.7 |
| 213 | Fusidic acid | 3 | 3000226 | 2 | 0.5 | 112.0 |
| 214 | Gabapentin | 3 | 3446 | 4 | 0.5 | 109.4 |
| 215 | Gentisic_acid | 3 | 3469 | 2 | 6 | 107.3 |
| 216 | Glucose | 3 | 5793 | 2 | 0.5 | 84.1 |
| 217 | Hydrocortisone | 3 | 5754 | 2 | 0.5 | 82.2 |
| 218 | Hydroquinine | 3 | 5318127 | 3 | 0.5 | 89.9 |
| 219 | Hydroxyzin | 3 | 3658 | 1 | 6 | 43.0 |
| 220 | Ibuprofen | 3 | 3672 | 2 | 0.5 | 63.3 |
| 221 | Idarubicin | 3 | 42890 | 4 | 0.5 | 68.9 |
| 222 | Imipramine | 3 | 3696 | 1 | 0.5 | 32.6 |
| 223 | Indomethacin | 3 | 3715 | 2 | 0.5 | 75.0 |
| 224 | Isoxsuprine | 3 | 3783 | 4 | 0.5 | 85.1 |
| 225 | L_Leucine_methyl_ester | 3 | 65105 | 1 | 0.5 | 76.7 |
| 226 | Leukomethylene_blue | 3 | 164695 | 3 | 48 | 57.3 |
| 227 | Loperamide | 3 | 3955 | 1 | 24 | 24.4 |
| 228 | Lynestrenol | 3 | 5857 | 2 | 6 | 110.3 |
| 229 | Mebhydrolin | 3 | 22530 | 1 | 0.5 | 41.9 |
| 230 | Meclofenamic acid | 3 | 4037 | 2 | 0.5 | 108.1 |
| 231 | Mefenamic acid | 3 | 4044 | 2 | 0.5 | 103.3 |
| 232 | Mepacrine | 3 | 237 | 3 | 0.5 | 44.3 |
| 233 | Mesoridazine | 3 | 4078 | 1 | 0.5 | 59.5 |
| 234 | Methapyrilene | 3 | 4098 | 1 | 0.5 | 96.2 |
| 235 | Metoclopramide | 3 | 4168 | 1 | 0.5 | 78.6 |
| 236 | Mianserin | 3 | 4184 | 1 | 0.5 | 111.6 |
| 237 | Mitotane | 3 | 4211 | 2 | 6 | 96.4 |
| 238 | Moexipril | 3 | 91270 | 4 | 0.5 | 99.7 |
| 239 | Montelukast | 3 | 5281040 | 2 | 24 | 120.5 |
| 240 | Moxisylyte | 3 | 4260 | 1 | 0.5 | 106.9 |
| 241 | Naphazoline | 3 | 4436 | 1 | 0.5 | 84.8 |
| 242 | Naproxen | 3 | 156391 | 2 | 0.5 | 72.0 |
| 243 | Nelfinavir | 3 | 64143 | 4 | 0.5 | 107.7 |
| 244 | Opipramol | 3 | 9417 | 3 | 0.5 | 61.3 |
| 245 | Oxeladin | 3 | 4619 | 1 | 0.5 | 75.2 |
| 246 | Oxolamine | 3 | 13738 | 1 | 0.5 | 82.8 |
| 247 | Paraxanthine | 3 | 4687 | 2 | 6 | 101.1 |
| 248 | Pergolide | 3 | 47811 | 2 | 6 | 69.2 |
| 249 | Perphenazine | 3 | 4748 | 3 | 0.5 | 20.1 |
| 250 | Phenothrin | 3 | 4767 | 2 | 6 | 102.2 |
| 251 | Phenserine | 3 | 192706 | 1 | 6 | 89.6 |
| 252 | Phentolamine | 3 | 5775 | 4 | 0.5 | 90.7 |
| 253 | Phenylmethylsulfonyl_fluoride | 3 | 4784 | 2 | 0.5 | 76.6 |
| 254 | Phenytoin | 3 | 1775 | 2 | 0.5 | 86.9 |
| 255 | Pirarubicin | 3 | 636397 | 4 | 0.5 | 101.2 |
| 256 | Pirenperone | 3 | 4847 | 3 | 0.5 | 117.4 |
| 257 | Pranlukast | 3 | 115100 | 2 | 0.5 | 123.9 |
| 258 | Profenamine | 3 | 3290 | 1 | 0.5 | 29.7 |
| 259 | Progesterone | 3 | 4920 | 2 | 0.5 | 103.5 |
| 260 | Propafenone | 3 | 4932 | 1 | 0.5 | 91.1 |
| 261 | Proparacaine | 3 | 4935 | 3 | 0.5 | 99.0 |
| 262 | Propranolol | 3 | 4946 | 1 | 0.5 | 92.3 |
| 263 | Putrescine | 3 | 1045 | 3 | 24 | 88.9 |
| 264 | Quetiapine | 3 | 5002 | 3 | 0.5 | 81.9 |
| 265 | Quinine | 3 | 8549 | 3 | 0.5 | 78.0 |
| 266 | Raloxifene | 3 | 5035 | 4 | 0.5 | 94.4 |
| 267 | Repaglinide | 3 | 4547 | 4 | 0.5 | 106.9 |
| 268 | Retinol | 3 | 445354 | 2 | 6 | 102.6 |
| 269 | Rifabutin | 3 | 6323490 | 4 | 0.5 | 120.5 |
| 270 | Rimantadine | 3 | 5071 | 1 | 0.5 | 70.3 |
| 271 | Rolipram | 3 | 5092 | 2 | 6 | 101.0 |
| 272 | Ropinirole | 3 | 5095 | 1 | 6 | 97.8 |
| 273 | Roxithromycin | 3 | 9567573 | 1 | 0.5 | 108.5 |
| 274 | S_Methylisothiourea | 3 | 5142 | 1 | 24 | 87.5 |
| 275 | Salmeterol | 3 | 5152 | 4 | 0.5 | 79.3 |
| 276 | SB-222200 | 3 | 3946663 | 2 | 24 | 116.4 |
| 277 | Sertindole | 3 | 60149 | 1 | 6 | 12.0 |
| 278 | Solasodine | 3 | 442985 | 1 | 24 | 22.2 |
| 279 | Sparteine | 3 | 644020 | 3 | 24 | 97.9 |
| 280 | Spiperone | 3 | 5265 | 1 | 0.5 | 72.6 |
| 281 | Spiramycin | 3 | 6440717 | 3 | 24 | 73.3 |
| 282 | Stanozolol | 3 | 25249 | 1 | 6 | 80.8 |
| 283 | Sulindac | 3 | 1548887 | 2 | 0.5 | 81.3 |
| 284 | Tacrine | 3 | 1935 | 1 | 0.5 | 90.2 |
| 285 | Tamoxifen | 3 | 2733526 | 1 | 6 | 4.1 |
| 286 | Telmisartan | 3 | 65999 | 4 | 0.5 | 114.7 |
| 287 | Thiocarlide | 3 | 3001386 | 2 | 24 | 77.4 |
| 288 | Tiagabine | 3 | 60648 | 4 | 0.5 | 95.1 |
| 289 | Tibolone | 3 | 444008 | 2 | 6 | 104.2 |
| 290 | Tirofiban | 3 | 60947 | 4 | 0.5 | 94.9 |
| 291 | Tomatidine | 3 | 65576 | 1 | 24 | 15.8 |
| 292 | Trifluoperazine | 3 | 5566 | 3 | 6 | 8.3 |
| 293 | Trifluperidol | 3 | 5567 | 1 | 0.5 | 74.0 |
| 294 | Tripelennamine | 3 | 5587 | 3 | 0.5 | 71.7 |
| 295 | Tulobuterol | 3 | 5606 | 1 | 0.5 | 90.9 |
| 296 | Uridine | 3 | 6029 | 2 | 0.5 | 90.8 |
| 297 | Venlafaxine | 3 | 5656 | 1 | 0.5 | 95.1 |
| 298 | Verapamil | 3 | 2520 | 1 | 0.5 | 79.6 |
| 299 | Vinblastine | 3 | 241903 | 3 | 6 | 70.3 |
| 300 | Vincamine | 3 | 5668 | 1 | 0.5 | 78.6 |
| 301 | Vincristine | 3 | 5978 | 3 | 0.5 | 107.0 |
| 302 | W_7 | 3 | 5681 | 1 | 0.5 | 65.4 |
| 303 | Warfarin | 3 | 6691 | 2 | 0.5 | 94.9 |
| 304 | Yohimbin | 3 | 8969 | 1 | 0.5 | 102.3 |
| 305 | Zafirlukast | 3 | 5717 | 2 | 0.5 | 89.6 |
| 306 | Zolantidine_(SKB41) | 3 | 91769 | 3 | 6 | 21.6 |
